# Unclassified Mixed Germ Cell-Sex Cord-Stromal Tumor of the Ovary: An Unusual Case Report

**DOI:** 10.7759/cureus.9350

**Published:** 2020-07-23

**Authors:** James Saenz, Juliana Rodriguez, María Beltran, Monica Medina, Rene Pareja

**Affiliations:** 1 Gynecologic Oncology, Instituto Nacional de Cancerologia, Universidad Militar Nueva Granada, Bogotá, COL; 2 Gynecologic Oncology, Fundación Santa Fe de Bogotá, Bogotá, COL; 3 Pathology, Instituto Nacional de Cancerologia, Universidad Militar Nueva Granada, Bogotá, COL; 4 Gynecologic Oncology, Clinica de Oncologia Astorga, Universidad Pontificia Bolivariana, Medellín, COL

**Keywords:** neoplasms, germ cell and embryonal, ovarian neoplasms, sex cord-gonadal stromal tumors

## Abstract

Unclassified mixed germ cell-sex cord-stromal tumor (UMGC-SCST) is a rare ovarian neoplasm composed of germ cells and sex cord elements, which occurs in genetically and phenotypically normal women without the usual histological features seen in gonadoblastoma. Few cases have been reported in the literature so far. The age of presentation is more frequent in girls younger than 10 years of age, although it can also occur in adult women. It can be associated with isosexual pseudoprecocity. The preferred management is the resection of the gonad that contains the tumor and the conservation of the opposite ovary and tube. This is a case of a 14-year-old patient, with precocious puberty and normal phenotype, diagnosed with this kind of ovarian tumor. A fertility preserving surgery with the resection of the right ovarian tumor and tube was performed. The patient was classified as stage IA according to the 2014 International Federation of Gynecology and Obstetrics (FIGO). She received adjuvant chemotherapy with bleomycin-etoposide-cisplatin for three cycles. After a follow-up of 24 months, she was found to be asymptomatic and free of relapse.

## Introduction

Within ovarian neoplasms, germ cell tumors constitute approximately 30% of all primary ovarian neoplasia, and up to 60% of all ovarian tumors in young patients can present this kind of tumor. One-third are malignant germ cell tumors. Most of them are pure, composed of a single cell line, but about 10% contain more than one component, of mixed germ cell histology, and have a combination with another cell line [1].

Unclassified mixed germ cell-sex cord-stromal tumor (UMGC-SCST) of the ovary is a very rare neoplasm with a germ tumor component and sex cord elements. Only 32 cases have been reported so far in the literature; its actual incidence is unknown. It is composed of germ cells and sex cord elements, appearing in genetically and phenotypically normal women without the histological morphology of a gonadoblastoma [2].

The aim of this article is to report an unusual case of a 14-year-old patient, with precocious puberty who was diagnosed with this kind of neoplasia.

## Case presentation

A 14-year old girl presented to the gynecology clinic in December of 2017 with a four-month history of abdominal pain and pelvic pressure sensation. As a relevant antecedent, her mother reported the appearance of the first menstruation in the girl at eight years of age, which corresponds to precocious puberty. As a comorbidity, she presented coarctation of the aorta with valvular heart disease (bicuspid aortic valve), with secondary arterial hypertension in pharmacological management. On physical examination, she presented an abdominopelvic mass of 20 cm, without other abnormal findings. Phenotypically, the patient appeared to be normal.

**Figure 1:**
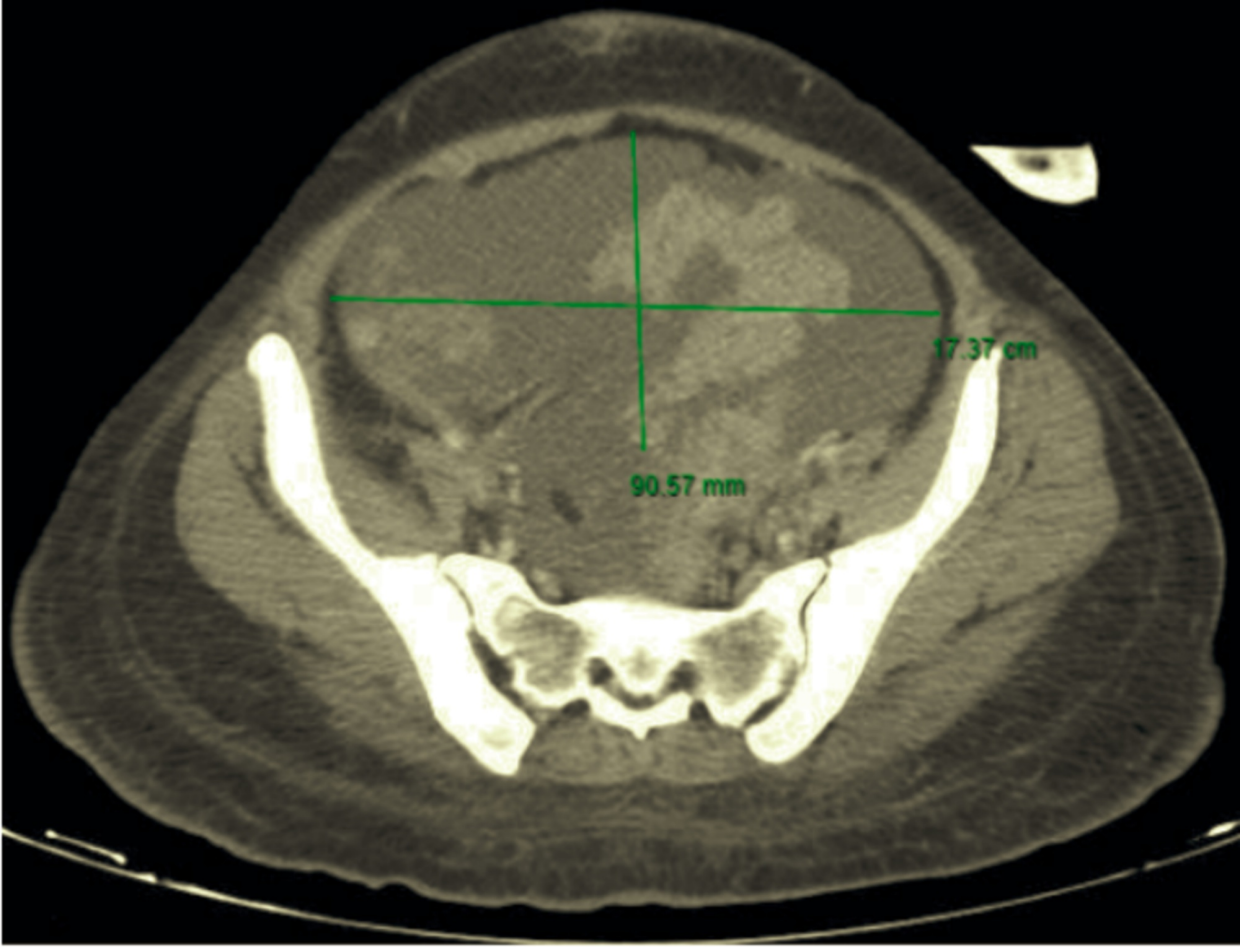
Computed axial tomography of the pelvis A solid abdominopelvic mass 17 x 9 x 21 cm in transverse, anteroposterior and vertical axes, respectively.

Computed axial tomography reported a solid abdominopelvic measuring 17 x 9 x 21 cm; she had no distant metastatic disease (Figure 1). An elevation in germ cell tumor markers was demonstrated: alpha-fetoprotein (AFP) > 1000 IU/mL (<400 IU/mL), lactate dehydrogenase (LDH) 15312 u/L (100-333 u/L), β-human chorionic gonadotropin (β-HCG) 20 U/mL (<5.0 U/mL), carbohydrate antigen 19-9 (CA 19-9) 16 U/mL (<120 U/mL), carcinoembryonic antigen (CEA) < 0.0 ug/L (0-3.8 ug/L).

Putting together the above-mentioned findings, conservative surgical management was offered, taking into account her age. She was taken to the operative room in December 2017, and resection of the right ovarian tumor, unilateral salpingectomy, and total omentectomy were done by laparotomy. The surgeon chose not to evaluate the regional lymph nodes, as there were no enlarged nodes. Surgery revealed a tumor depends on the right ovary, with a 24 cm, intact capsule, that weighed 2460 g. No intraoperative rupture was recorded. No ascites, tumor implants, or disease other than described was seen in the rest of the cavity. Pelvic lavage and diaphragmatic cytology were taken with negative results.

**Figure 2:**
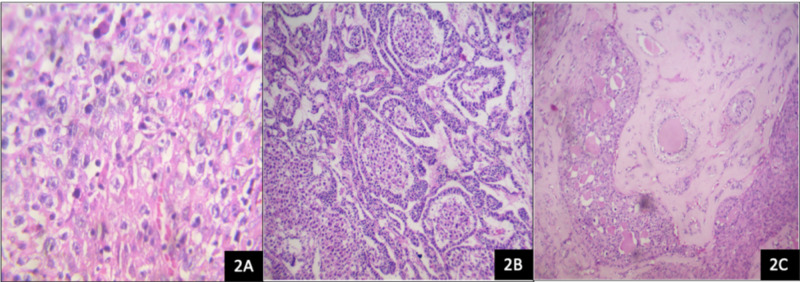
Ovarian mixed germ cell-sex cord-stromal tumor, unclassified H&E, hematoxylin and eosin. (2A) Dysgerminoma component with energetic mitotic activity (H&E, 10x). (2B) Component of yolk sac tumor channels forming microcysts lined by atypical epithelial cells (H&E, 10x). (2C) Component of sex cord with annular tubules (H&E, 10x).

**Figure 3:**
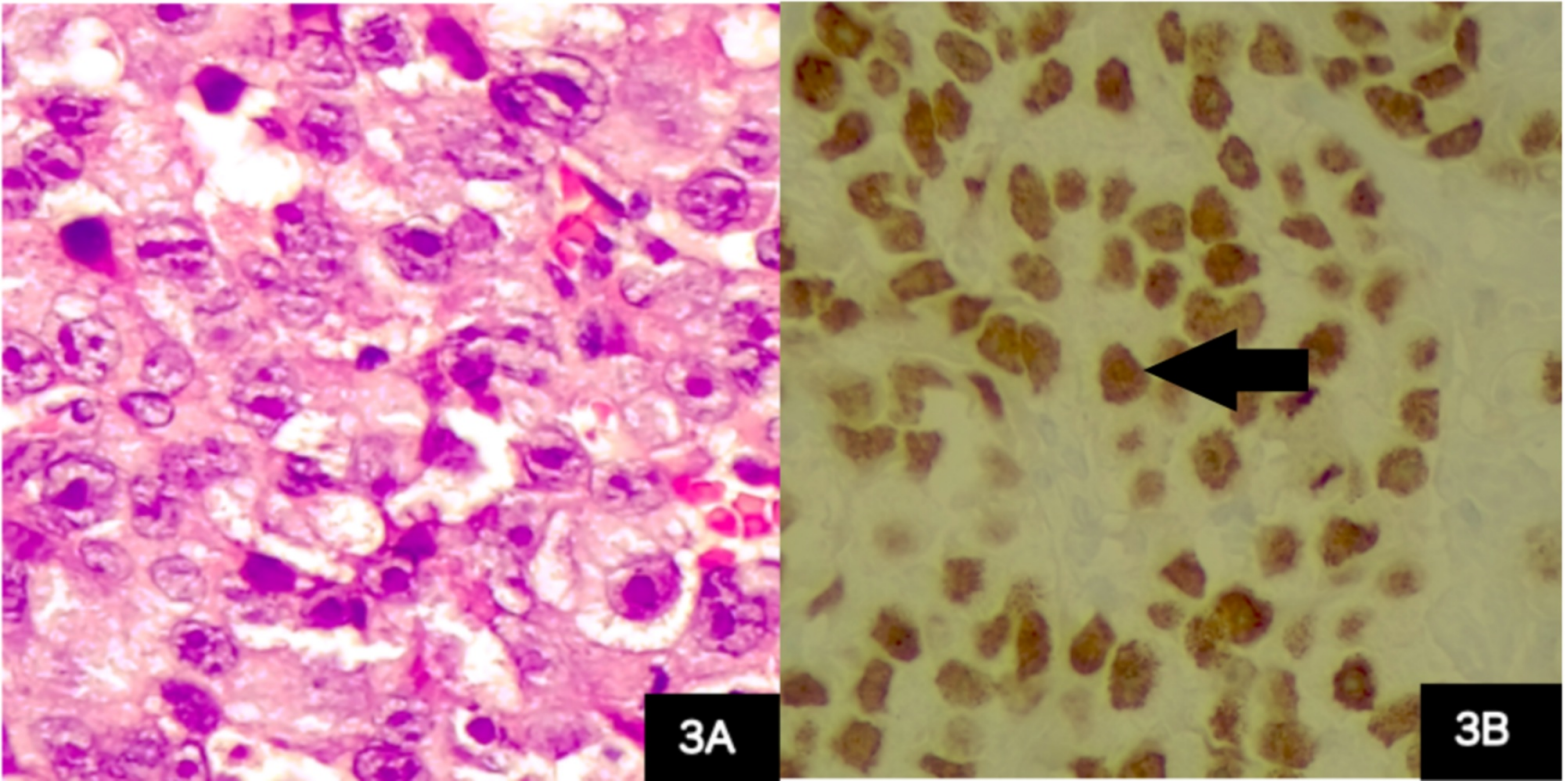
Ovarian tumor, dysgerminoma component (3A) Dysgerminoma component, abundant clear or slightly eosinophilic cytoplasm, large round nuclei with prominent nucleoli (hematoxylin and eosin stain, 40x). (3B) Positive immunohistochemistry for Octamer-binding transcription factor 4, expressed in germ cells (black arrow).

**Figure 4:**
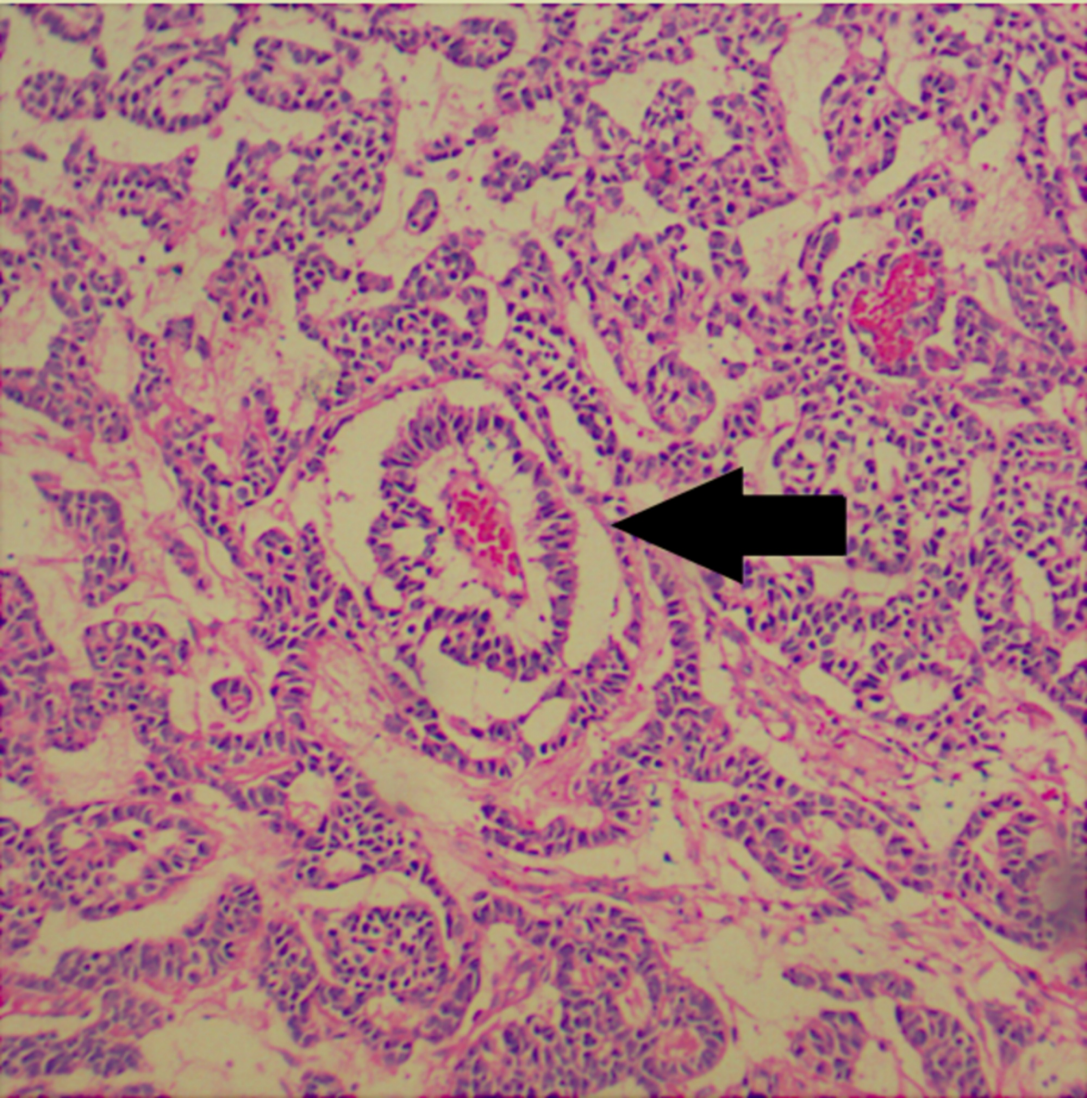
Ovarian tumor, component of yolk sac Focally, it resembles Schiller-Duval body-type fibrovascular structures (black arrow). Hematoxylin and eosin stain, 40x.

**Figure 5:**
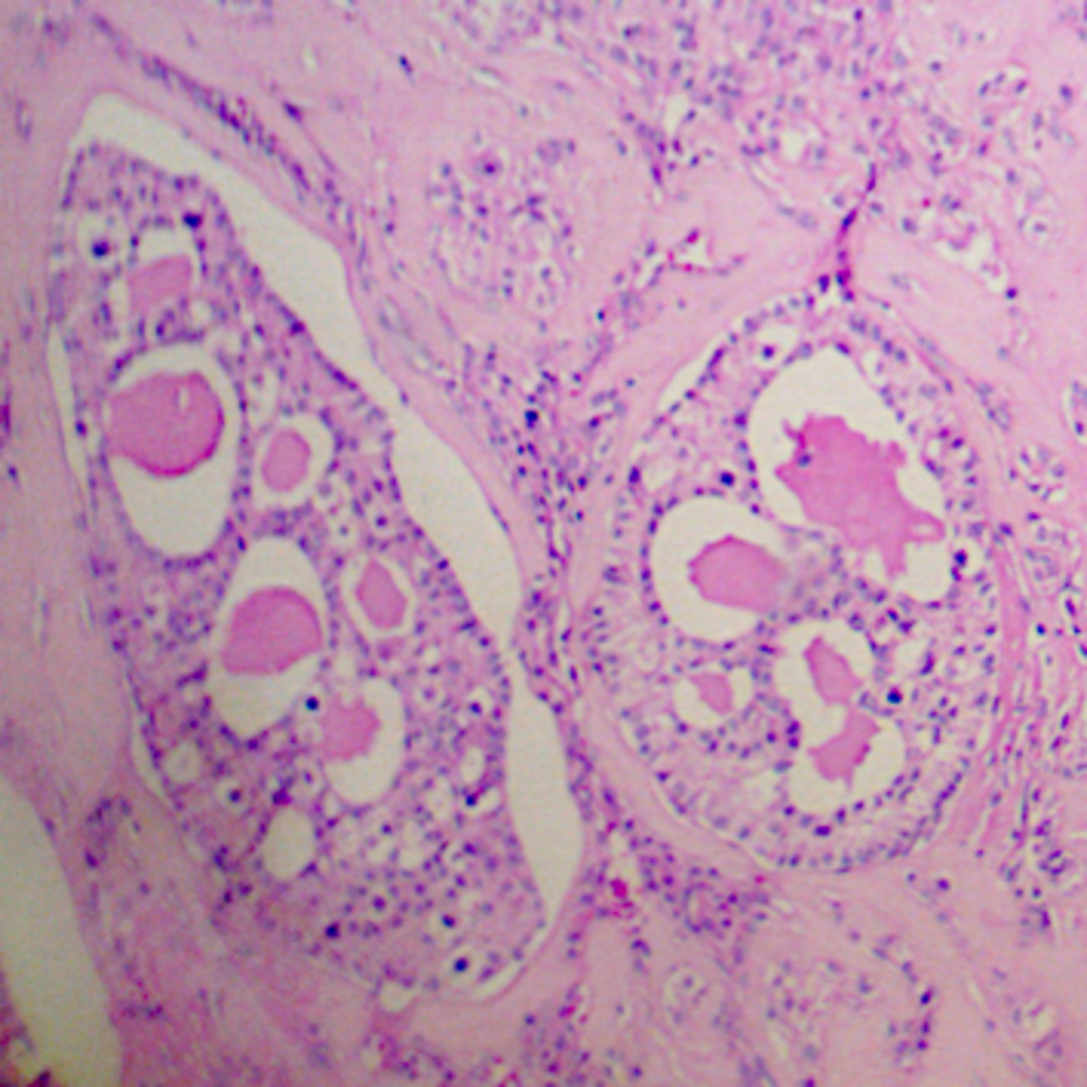
Ovarian tumor, component of sex cord with annular tubules Reverse polarity is recognized. Hematoxylin and eosin stain, 40x.

The histological report showed a mixed germ cell-sex cord-stromal tumor, unclassified: dysgerminoma and yolk sac tumor (70%) and sex cord with annular tubules (30%) (Figure 2). The dysgerminoma component contains large immature cells, a large clear nucleus, with a notorious nucleolus (Figure 3). The component of the yolk sac resembled Schiller-Duval body-type fibrovascular structures (Figure 4). In the component of sex cord with annular tubules, reverse polarity was recognized (Figure 5). The tumor growth pattern was diffuse. Immunohistochemistry was positive for dysgerminoma, placental alkaline phosphatase (PLAP), CD117 (c-kit), Octamer-binding transcription factor 3/4 (OCT3/4), Sal-like protein 4 (SALL4); for the yolk sac AFP, SALL4; and for the sex cord, Inhibin, cytokeratin (CK), calretinin. Synaptophysin and chromogranin were negative.

The patient was staged according to the 2014 International Federation of Gynecology and Obstetrics (FIGO) classification as IA. Adjuvant chemotherapy with a bleomycin-etoposide-cisplatin (BEP) scheme was recommended for three cycles that started in January 2018 and were completed in March of the same year.

Serum tumor markers were measured after completing the adjuvant treatment, showing a significative decrease: Serum AFP 2.1 IU/mL (<400 IU/mL), LDH 459 u/L (100-333 u/L), β-HCG <1.0 U/mL (<5.0 U/mL). Clinical follow-up was performed with physical examination every three months and computed axial tomography of the abdominopelvic region every six months for two years without evidence of relapse. Menses were normal during follow-up. The last follow-up was done 24 months after treatment, and the patient was asymptomatic and free of disease.

## Discussion

Unclassified mixed germ cell-sex cord-stromal tumor was initially described by Talerman in 1972 [3]. This kind of tumor is very rare; most cases are found in infants and children younger than 10 years of age, although there are also cases described in adult women. We present this case with a satisfactory oncologic result two years after its treatment.

MGC-SCSTs show germ cell and sex cord components. They are divided into two groups: gonadoblastoma, including gonadoblastoma with a malignant germ cell tumor, and UMGC-SCST [4].

The histogenesis of ovarian UMGC-SCST has not been clearly established. It has been proposed that it can originate from the simultaneous transformation of germ cells and sex cord derivatives, sometimes with the formation of imperfect follicular structures. Roth and Cheng describe 12 cases originating from the ovary [5].

Macroscopically, this tumor appears as a large, unilateral solid mass. At the microscopic level, germ cells resemble dysgerminoma cells and are immunoreactive for PLAP, OCT4, and c-kit. The sex cord elements may form cords or trabeculae, hollow or solid tubules, cysts, or grow diffusely and are immunoreactive for inhibin [6].

Approximately 10% of these tumors have malignant germ cell components compared to 60% of gonadoblastomas. The tumor differs from gonadoblastoma in its macroscopic appearance, histological pattern, absence of regressive changes, and occurrence in normal gonads of phenotypic and genetically normal women (Table 1) [4,7].

**Table 1:** Characteristics of gonadoblastoma and unclassified mixed germ cell-sex cord-stromal tumor

	Gonadoblastoma	Mixed germ cell-sex cord-stromal tumor, unclassified
Age	10-30 years	<10 years
Karyotype	Abnormal	Normal 46 XX
Phenotype	Symptoms virilization	Normal female
Macroscopic features	<3 cm bilateral, calcifications	Large, unilateral tumors
Microscopic features	Variable nesting, hyaline bodies, calcifications, stromal luteinization, 60% mixed	Architecture variable, hyaline bodies, stromal luteinization rare

Although chromosomal abnormalities or gonadal dysgenesis is generally not present, Speleman et al. report one case of monosomy 22 [8].

Roth and Cheng propose criteria to differentiate the UMGC-SCST: a diffuse growth pattern, lacks rounded islands or nests, lacks basal membranes or calcifications, and also contains germ cells that are generally malignant in tumors originating from the ovary [9].

Regarding the clinical characteristics, most have been found in post-pubertal women, although cases have been reported in girls under 10 years of age. In these women, approximately 80% have a concomitant malignancy of germ cells [4]. Symptoms include abdominal pain, abnormal uterine bleeding, ascites, and abdominal or pelvic masses. Pang et al. reported the first case associated with embryonal carcinoma components [10].

They are a less frequent cause of sexual precocity in girls, but it should be considered as a differential diagnosis in the etiology of the latter. Lacson et al. described two cases with isosexual precocity, and the presence of metastasis [11]. The patient had undergone precocious puberty, which had not been studied, and during her adolescence presented the abdominopelvic mass leading to the diagnosis. The diagnostic approach includes images to characterize adnexal tumor lesions as in other ovarian neoplasms.

For young patients with an adnexal mass, tumor markers oriented to germ neoplasms should be requested. In the reported cases of this neoplasm, the elevation of AFP, LDH, and β-HCG has been mentioned [12].

The proposed management for this neoplasm is surgical. Because the mixed germ cell tumor is generally unilateral, resection of the macroscopically normal-looking contralateral ovary should not be performed. In addition, since these are young patients with a desire for fertility, conservative surgical management has been reported in the cases described [13].

Platinum-based chemotherapy has improved the prognosis for these patients with diagnosis of secondary germ cell malignancies. Adjuvant treatment is proposed as in germ cell neoplasms. The patient in this case received adjuvant therapy with a BEP scheme for the germline tumor component, with a complete oncological response and without limiting toxicity [4,14].

The oncological prognosis is favorable. In three cases, the patients presented a normal pregnancy after treatment. Disease-free periods ranged from one to 15 years, and only three cases reported recurrence or metastasis [4,10,11]. Currently, the patient is 16-years-old and is in a disease-free period.

## Conclusions

In conclusion, this is a very rare neoplasm, the management being the resection of the gonad that contains the tumor and the conservation of the opposite side gonad that is normal. It should be considered in the differential diagnosis of a female with an abdominal mass and precocious puberty.
